# Differentiating between cardiac amyloidosis and hypertrophic cardiomyopathy on non-contrast cine-magnetic resonance images using machine learning-based radiomics

**DOI:** 10.3389/fcvm.2022.1001269

**Published:** 2022-10-26

**Authors:** Shu Jiang, Lianlian Zhang, Jia Wang, Xia Li, Su Hu, Yigang Fu, Xin Wang, Shaowei Hao, Chunhong Hu

**Affiliations:** ^1^Department of Radiology, The First Affiliated Hospital of Soochow University, Suzhou, China; ^2^Department of Radiology, The Yancheng Clinical College of Xuzhou Medical University and The First People’s Hospital of Yancheng, Yancheng, China; ^3^Department of Ultrasound, The Yancheng Clinical College of Xuzhou Medical University and The First People’s Hospital of Yancheng, Yancheng, China; ^4^Department of Radiology, Northern Jiangsu People’s Hospital, Yangzhou, China; ^5^Department of General Medicine, The Sixth Affiliated Hospital of Nantong University, Yancheng Third People’s Hospital, Yancheng, China; ^6^Department of Clinical Nutrition, The Sixth Affiliated Hospital of Nantong University, Yancheng Third People’s Hospital, Yancheng, China; ^7^Siemens Healthineers Digital Technology (Shanghai) Co., Ltd., Shanghai, China

**Keywords:** machine learning, texture analysis, cardiac amyloidosis, cardiac magnetic resonance, hypertrophic cardiomyopathy, non-contrast

## Abstract

**Objectives:**

This study aimed to determine whether texture analysis (TA) and machine learning-based classifications can be applied in differential diagnosis of cardiac amyloidosis (CA) and hypertrophic cardiomyopathy (HCM) using non-contrast cine cardiac magnetic resonance (CMR) images.

**Methods:**

In this institutional review board-approved study, we consecutively enrolled 167 patients with CA (*n* = 85), HCM (*n* = 82), and 84 patients with normal CMR served as controls. All cases were randomized into training [119 patients (70%)] and validation [48 patients (30%)] groups. A total of 275 texture features were extracted from cine images. Based on regression analysis with the least absolute shrinkage and selection operator (LASSO), nine machine learning models were established and their diagnostic performance determined.

**Results:**

Nineteen radiomics texture features derived from cine images were used to differentiate CA and HCM. In the validation cohort, the support vector machine (SVM), which had an accuracy of 0.85, showed the best performance (MCC = 0.637). Gray level non-uniformity (GLevNonU) was the single most effective feature. The combined model of radiomics texture features and conventional MR metrics had superior discriminatory performance (AUC = 0.89) over conventional MR metrics model (AUC = 0.79). Moreover, results showed that GLevNonU levels in HCM patients were significantly higher compared with levels in CA patients and control groups (*P* < 0.001). A cut-off of GLevNonU ≥ 25 was shown to differentiate between CA and HCM patients, with an area under the curve (AUC) of 0.86 (CI:0.804–0.920). Multiple comparisons tests showed that GLevNonU was significantly greater in LGE+, relative to LGE-patient groups (CA+ vs. CA- and HCM+ vs. HCM-, *P* = 0.01, 0.001, respectively).

**Conclusion:**

Machine learning-based classifiers can accurately differentiate between CA and HCM on non-contrast cine images. The radiomics-MR combined model can be used to improve the discriminatory performance. TA may be used to assess myocardial microstructure changes that occur during different stages of cardiomyopathies.

## Introduction

Cardiac amyloidosis (CA) and hypertrophic cardiomyopathy (HCM) are associated with increased left ventricular wall thickness (LVWT). Differential diagnosis of CA and HCM is still challenging in some instances in which cardiovascular magnetic resonance (CMR) plays an essential role. Cine sequences provide the morphological and functional characterization of the heart. Still, the differential diagnosis of amyloidosis is typically based on keypoint information from late gadolinium enhancement (LGE) and mapping technique (1, 2) combined with clinical information. Mapping is an important novel technique, but these sequences are not universally available to date. Gadolinium administration can cause nephrogenic systemic sclerosis in patients with impaired renal function. In addition, there is evidence that gadolinium may be deposited in brain regions in humans (3, 4). This calls for the development of gadolinium-free techniques to facilitate myocardial fibrosis characterization.

In cardiac amyloidosis (CA), fibrillar proteins are extracellularly deposited, disrupting normal tissue architecture and function (5). Compared with CA, HCM is relatively more common, and is thought to be caused by a gene mutation that encodes the sarcomere. Histopathological findings in HCM include hypertrophy and disarray of cardiomyocytes as well as interstitial fibrosis (6).

Pathological changes lead to signal abnormalities. In the context of a purely visual, subjective analysis, it is impossible to discern signal abnormalities that are visible on LGE imaging and invisible on non-contrast cine. The radiomics is a non-invasive way of analyzing and calculating the shape and textural information in radiological images. Recently, machine learning have been used to deal with the level of information produced by radiomics feature extraction and identify new patterns in large datasets (7, 8). Such tools provide objective assessment of organ heterogeneity and lesions, thereby revealing important details in the tissue microenvironment. It is increasingly used in cardiovascular diseases and across a range of disciplines. Myocardium heterogeneity can be assessed objectively using ML beyond subjective visual interpretations, and subtle changes in myocardial microstructure may be detected during cardiomyopathy (9). Machine learning algorithms are classified as supervised or unsupervised. These algorithms can deal with many quantitative variables of radiomics to characterize tissues. The goal of supervised learning is to identify unknown patterns from datasets using algorithms based on a subset of a trained data set with known labels. There are several clinical applications for CMR-based ML, irrespective of the methodology used (10–13). For instance, through texture analysis (TA) with Boruta machine learning algorithms (10), CMR cine images have been used to differentiate between HCM and control subjects. Using quantitative CMR images is another approach to TA in CMR imaging (11, 12). Hence, we hypothesized that a radiomics approach could identify differences in the myocardial texture of CA and HCM on CMR cine images.

In this study, we aimed to determine whether ML-based classifications incorporating TA can be applied in the differential diagnosis of CA and HCM using non-contrast cine images.

## Materials and methods

### Patient population

We recruited 251 consecutive patients subjected to routine CMR examination from June 2017 to January 2022. Inclusion criteria for patients (CA and HCM patients) and control group were based on an established diagnostic criteria and CMR measurements (14, 15) (Supplementary Methods).

All patients with clinically suspected systemic amyloidosis and histologically confirmed systemic light chain (AL) amyloidosis by Congo red and immunohistochemical diagnosis were subjected to CMR. Cardiac amyloidosis was defined by the combination of typical CMR findings and biopsy-proven AL amyloidosis on cardiac or non-cardiac tissues. Patients enrolled in this study participate in our ongoing bidirectional cohort study of cardiac amyloidosis. Hence, this was a secondary analysis of the cohort study based on radiomics. Finally, 85 patients were recruited.

The HCM was diagnosed by either unexplained LVH (LVWT ≥ 15 mm) or having a sarcomeric mutation (genotype positive phenotype negative, G+ P−, *n* = 11) that causes HCM (16). Those with previous septal ablation or myectomy were excluded. In total, 82 patients who satisfied the diagnostic criteria were enrolled in this study.

**FIGURE 1:**
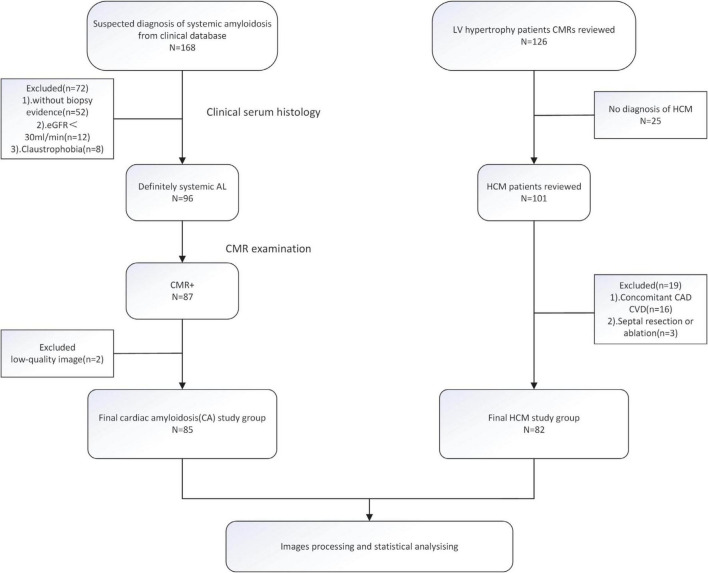
The flowchart of inclusion and exclusion criteria.

The control group consisted of 7 healthy adult subjects recruited through advertisement and 77 clinical patients screened for any history of cardiac disease who did not exhibit cardiovascular disease or diabetes symptoms, including cardiac surgery or interventions. Finally, a total of 84 subjects were enrolled in this study as control (Supplementary Methods). The patient enrollment procedure is shown in Figure 1.

The CA and HCM groups were randomized into training and validation cohorts in a 7:3 ratio (Supplementary Figure 1).

This study was approved by the Medical Ethics Committee of our institution. Prior to their recruitment, participants were required to provide a written informed consent.

### Cardiac magnetic resonance imaging data acquisition

ECG-gated CMR imaging was performed using a 3.0 T whole body MR scanner (MAGNETOM Skyra, Siemens Healthcare, Erlangen, Germany). Cine images were obtained by a 2D steady-state precession (SSFP) sequence before administration of contrast medium (Gd-DTPA, 0.02 mmol/kg). LGE images were acquired 10 min after contrast injection using a segmented-phase sensitive inversion recovery gradient echo (PSIR-GRE) sequence.

### Cardiac magnetic resonance imaging data analysis and patient subgroups

An analysis of left ventricular volume was performed on short-axis (SAX) cine images using commercially available software (cvi42, Circle, Cardiovascular Imaging, Calgary, Alberta, Canada).

Furthermore, some patients (equal LVWT subgroup) were matched with respect to potential cofounding factors to allow accurate evaluation of the test’s diagnostic significance in patients with similar morphological features. Therefore, HCM patients with high clinical suspicion (LVWT > 13 mm and genotype positive) and CA patients (LVWT ≥ 15 mm) were matched strictly by gender, maximal LVWT and age were assigned to the similar asymmetric septal hypertrophy (ASH: was defined as septal to posterior free wall ratio > 1.3) group (6, 17) (Supplementary Methods and Supplementary Table 1).

In further analyses, we compared HCM and CA patients from the feature selection dataset with control participants to reveal detailed tissue characteristics specific to the disease.

The LGE image evaluation procedure was performed by two blinded readers (one had 3 years of experience in cardiovascular imaging while the senior radiologist had 6 years of experience). Patients were assigned into two subgroups based on whether they had visual LGE (LGE+) or not (LGE−). In case of differences in opinion, a consensus was reached by discussion.

### Placement of region of interest

**FIGURE 2:**
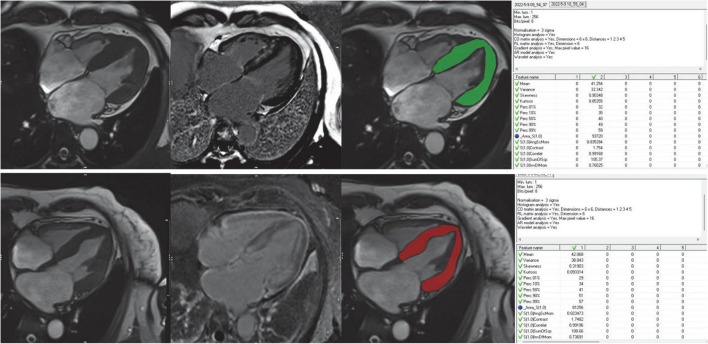
Workflow for image analysis including TA. Cine images were used for visual wall motion analysis and functional assessment LGE images were used to further divide the patient cohort into LGE+ and LGE- subjects for subgroup analyses. Freehand ROIs were drawn encompassing the entire myocardium while carefully excluding the endo- and epicardial regions. The rightmost column showed the feature extraction of the ROIs using the MaZda software.

The region of interest (ROIs) covering the left ventricular myocardium was manually drawn by two radiologists using the MaZda software package (version 4.6.0)^1^ on 4 chamber long-axis (LAX) image in the end-systolic frame (shown in Figure 2). The two radiologists were both blinded to clinical results. Trabeculated and epicardial borders were excluded to eliminate partial volume effects. Unlike most previous studies, we innovated by choosing 4 chamber LAX image in the end-systolic images, however, most previous studies choosed SAX end-diastolic images (Supplementary Methods contains the reason details for placement of ROIs).

### Radiomics extraction and analysis

Cine sequences were acquired in accordance with the guidelines of the Society for CMR (SCMR) and retrieved from Picture Archiving and Communication System (PACS) of our institution with the corresponding window width and position. For each patient, trigger time was visually chosen based on smallest LV dimension (end-systolic).

Gray-scale normalization was performed between the mean and three standard deviations (“± 3σ” method) before feature extraction. In accordance with previous studies, it minimizes the effects of inter-scanner differences and improves the reproducibility as well as robustness of radiomics features (7, 18). A total of 275 radiomics features within each ROI were separately extracted from 5 subsets of image descriptors (Table 1). MaZda’s official website contains a detailed description of these radiomics features and their mathematical formula.

**TABLE 1:** Overview of all computed texture categories with corresponding features and selected features for classification.

Texture category	Texture feature	Classification between CA and HCM	Number of selected features
**Histogram**	Mean, variance, skewness, kurtosis, percentiles (1, 10, 50, 90, 99%)	percentiles (1, 10%)	2
**Co-occurrence matrix** (computed for four directions [(a,0), (0,a), (a,a), (0,-a)] at five interpixel distances (a = 1–5))	Angular second moment, contrast, correlation, entropy, sum entropy, sum of squares, sum average, sum variance, inverse different moment, difference entropy, difference variance	S(1,1) Contrast S(2,0) DifVarnc S(0,2) SumAverg S(0,2) SumVarnc S(0,3) SumVarnc S(3,3) SumAverg S(4,-4) AngScMom S(0,5) InvDfMom S(0,5) SumVarnc S(5,5) SumOfSqs S(5,-5) Contrast S(5,-5) InvDfMom	12
**Run-length matrix** [computed for four angles (vertical, horizontal, 0°, and 135°)]	run-length non-uniformity, gray-level non-uniformity, long run emphasis, short run emphasis, fraction of image in runs	GLevNonU	1
**Absolute gradient**	mean, variance, skewness, kurtosis, and non-zeros 5		
**Autoregressive model**	Teta 1 to 4, sigma	Teta1 Sigma	2
**Wavelet transform** (calculated for four subsampling factors (*n* = 1–4)	Energy of wavelet coefficients in low-frequency sub-bands, horizontal high-frequency	WavEnHL_s.4 WavEnHH_s.4	2
	sub-bands, vertical high-frequency sub-bands, and diagonal high-frequency sub-bands		

### Analysis of feature reproducibility and feature selection

All extracted radiomics features were evaluated by calculating intra-class correlation coefficients (ICC). Calculations for the ICC were generated using the “icc” command in the irr package in R. For further analysis, reproducibility was rated as excellent only for features with an ICC value of 0.75 (Supplementary Figure 2).

Then, the least absolute shrinkage and selection operator (LASSO) regression was applied to each variable classifier in the training cohort, according to binomial deviance minimization criteria. An unbiased analysis was produced and cross-validated 10 times to prevent overfitting (Supplementary Figure 3).

### Classification and validation

In this study, six supervised machine learning algorithms were utilized: K-Nearest Neighbor (KNN), Random Forest (RF), Naïve Bayes (NB), Support-Vector Machine (SVM), Logistic Regression (LR), and Artificial Neural Networks (ANN). Nine machine-learning classifiers were constructed in the training cohort, which were tested in the validation cohort. Based on previous studies, we calculated the Matthews correlation coefficient (MCC, Eq. 1) of the confusion matrix to quantify how robust the model is in imbalanced data (7, 19). Additionally, specificity, sensitivity, and accuracy were calculated.


$$MCC=\left[\left(TP\times TN\right)-\left(FP\times FN\right)\right]/[\left(TP+FP\right)\left(TP+FN\right)\left(TN+FP\right)\left(TN+FN\right)]{}^{1/2}$$

The equation of MCC; MCC, Matthews correlation coefficient; TP, true positive; TN, true negative; FP, false positive, FN, false negative.

### Statistical analysis

Statistical analyses were performed using R statistical software (version 3.3.3) and IBM SPSS (version 26.0). Kolmogorov-Smirnov was used to determine whether data was normally distributed. Categorical data are presented as percentages, continuous and normally distributed data are expressed as mean ± standard deviation (SD) while data that did not conform to normal distribution were expressed as medians and interquartile ranges (IQR). The independent *t*-tests or Mann-Whitney *U*-tests were used to compare continuous variables while categorical variables between groups were compared using the Chi-square tests. Multiple non-parametric comparisons between subgroups and controls were conducted using the Kruskal-Wallis tests. False Discovery Rate (FDR) was calculated using the Benjamini-Hochberg’s (BH) method. *P* ≤ 0.05 was the threshold for statistical significance. The areas under the receiving operating characteristic (ROC) curves were used to evaluate the accuracy of the three models for differential diagnosis. Comparing the different AUCs was done using DeLong’s test.

## Results

### Patient population

A total of 167 patients (CA85, HCM82) and 84 control subjects were enrolled in this study; Their demographic and LV volumetric data is presented in Table 2. There were significant differences between groups in terms of Maximum LVWT, Asymmetric septal hypertrophy, LV mass, and LGE presence.

**TABLE 2:** Patient characteristics and morphological, functional measures based on cardiovascular magnetic resonance.

Parameter	CA patients (*n* = 85)	HCM patients (*n* = 82)	Control (*n* = 84)	*P*-value	*P* *	*P* **	*P* ***
Age (year)	54 ± 9	46 ± 14	45 ± 8	0.56	0.39	0.32	0.91
Male, *n* (%)	40 (47)	44 (54)	51 (61)	0.20	0.39	0.09	0.36
BMI (kg/m^2^)	23.7 ± 3.2	25.5 ± 3.2	24.0 ± 3.5	0.78	0.53	0.93	0.90
SBP (mmHg)	116.6 ± 17.3	132.6 ± 22.5	116.8 ± 11.7	0.001	0.001	0.93	0.001
DBP (mmHg)	71.1 ± 11.0	82.04 ± 14.7	79.4 ± 8.4	0.08	0.03	0.08	0.58
NYHA II-III stage, *n* (%)	47 (55)	23 (28)	0 (0)	−	0.001	−	−
Maximum LVWT (mm)	16.4 ± 4.5	20.1 ± 5.1	9.3 ± 2.4	0.01	0.01	0.01	0.01
Asymmetric septal hypertrophy, *n* (%)	42 (49)	55 (67)	0 (0)	−	0.03	−	−
LVEDV (ml/m^2^)	55.2 ± 12.2	71.7 ± 20.1	74.6 ± 17.7	0.01	0.01	0.01	0.48
LVESV (ml/m^2^)	24.6 ± 11.6	23.6 ± 12.1	22.7 ± 8.2	0.29	0.75	0.36	0.79
LVEF (%)	63.9 ± 13.0	68.3 ± 7.1	62.8 ± 8.4	0.07	0.09	0.62	0.02
LV mass (g/m^2^)	94.5 ± 29.2	111.7 ± 31.5	49.0 ± 12.5	0.01	0.01	0.001	0.001
LGE presence, *n* (%)	68 (80)	51 (62)	0 (0)	−	0.02	−	−

CA, cardiac amyloidosis; HCM, hypertrophic cardiomyopathy; BMI, body mass index; SBP, systolic blood pressure; DBP, Diastolic blood pressure; NYHA, New York Heart Association; LVWT, Left ventricular wall thickness; LVEDV, left ventricular end-diastolic volume; LVESV, left ventricular end-systolic volume; LVEF, left ventricular ejection fraction; LVmass, left ventricular mass; LGE, Late gadolinium enhancement. Values are given as mean ± standard deviation for continuous variables; and count (%) for categorical variables. P-value, p-value of ANOVA; *CA vs. HCM; **CA vs. Controls; ***HCM vs. Controls.

### Diagnostic performance of conventional magnetic resonance imaging model

Four conventional MR metrics were used in the model to classify CA and HCM: Maximum LVWT, asymmetric septal hypertrophy, LV mass, and LGE presence. The AUC, sensitivity, and specificity of the model were 0.79 (CI: 0.72–0.86), 0.81 (CI: 0.72–0.88), 0.79 (CI: 0.69–0.86) in the validation group, respectively.

### Radiomics features selection and diagnostic performance of radiomics model

**FIGURE 3:**
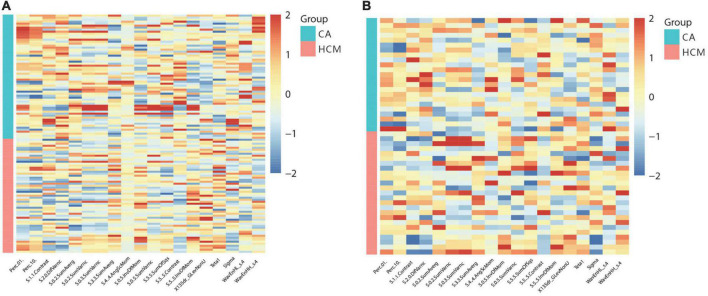
Heat-maps of the selected features from cine for the train **(A)** and validation **(B)** cohort, which were plotted in red (HCM, hypertrophic cardiomyopathy) vs. blue (CA, cardiac amyloidosis) color scales to show the distribution and differences of normalized (z-score) feature values.

In this study, 265 of 275 radiomics features showed excellent reproducibility (ICC ≥ 0.75). For differentiation of CA from HCM, 19 features in cine images were generated using the LASSO feature selection analysis. The selected features and their values are presented in Table 1 and Figure 3. Auto- and cross-correlations of the 19 features are illustrated in this correlogram (Supplementary Figure 4).

**FIGURE 4:**
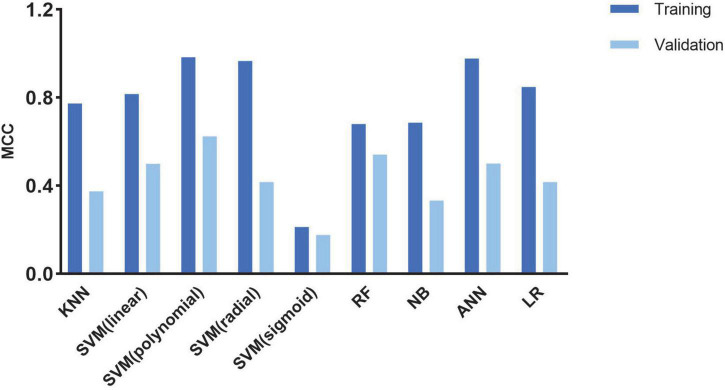
Histogram shows the performance of classifiers from cine images for discriminating CA and HCM in the train and validation cohort.

A cross-validation-trained SVM polynomial classifier can achieve an MCC of 0.98 and an accuracy of 0.99 (CI: 0.97–1.00). In the validation cohort, the SVM polynomial classifier yielded the highest MCC and accuracy scores: 0.637 and 0.852 (CI: 0.76–0.91) (Table 3 and Figure 4).

**TABLE 3:** Classification results of machine learning–based classifiers in differentiating CA and HCM.

Classifier	Train cohort				Validation cohort			
	ACC	SEN	SPE	MCC	ACC	SEN	SPE	MCC
KNN	0.882	0.947	0.823	0.773	0.688	0.76	0.609	0.374
SVM (linear)	0.908	0.86	0.952	0.817	0.75	0.76	0.739	0.499
SVM (polynomial)	0.992	1	0.983	0.983	0.852	0.855	0.850	0.637
SVM (radial)	0.983	0.982	0.984	0.966	0.708	0.72	0.696	0.416
SVM (sigmoid)	0.605	0.632	0.581	0.212	0.604	0.56	0.652	0.176
RF	0.84	0.807	0.871	0.68	0.771	0.8	0.739	0.541
NB	0.84	0.895	0.79	0.686	0.667	0.68	0.652	0.332
ANN	0.983	0.966	1	0.977	0.75	0.741	0.762	0.5
LR	0.924	0.936	0.912	0.848	0.708	0.696	0.72	0.416

ANN, artificial neural network; RF, random forest; SVM, support vector machine; NB, Naive Bayesian; KNN, K-nearest neighbor; LR, Logistic Regression; ACC, accuracy; SEN, sensitivity; SPE, specificity; MCC, Matthews correlation coefficient.

### Radiomics features for identifying myocardial tissue alterations in cardiac amyloidosis and hypertrophic cardiomyopathy patients

After the multi-part dimension reduction process, analysis of CA (*n* = 85) and HCM (*n* = 82) patients revealed significant differences in 5 out of the19 radiomics features, including 3 s-order features (Gray level non-uniformity, GLevNonU; Gray-level run-length matrix, GLRLM) (DifVarnc, Contrast; Gray-level co-occurrence matrix, GLCM) and 2 higher-order metrics features (Teta1, Sigma). Multiple logistic regression analyses revealed that GLevNonU was the best single texture feature for discriminating between HCM and CA patients. In ROC analyses, AUC was 0.86 (CI: 0.80–0.92) with GLevNonU ≥ 25 as the optimal cut-off, where higher values indicated a higher likelihood for HCM. GlevNonU had a sensitivity of 0.68 (CI: 0.43–0.86) and a specificity of 0.74 (CI: 0.51, 0.89) with a diagnostic accuracy of 0.71 (CI: 0.56–0.83) in equal LVWT and ASH subgroups (Supplementary Figure 5).

**FIGURE 5:**
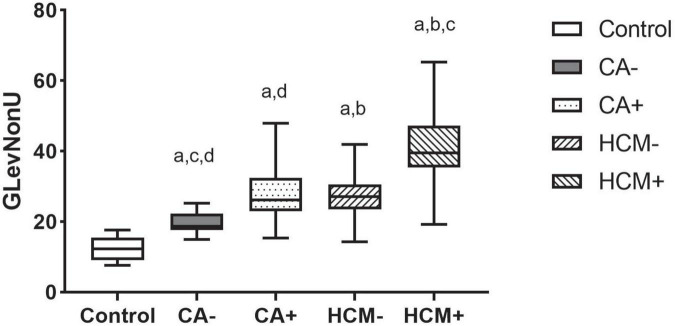
Boxplot indicating median and interquartile ranges for the GLevNonU feature for each subgroup. All Values are Median +/-interquartile. All results were analyzed using the Kruskal-Wallis test followed by Benjamini-Hochberg’s multiple-testing corrections. ^a^*p* < 0.05 compared to control. ^b^*p* < 0.05 compared to CA_*LGE*–_. ^c^*p* < 0.05 compared to CA_*LGE*+_. ^d^*p* < 0.05 compared to HCM_*LGE*–_.

CA and HCM patients were, respectively, assigned into LGE positive (i.e., CA+, HCM+) and LGE negative (i.e., CA−, HCM-) groups, and compared with controls. In subgroup analysis, pairwise comparisons revealed that each of these subgroups significantly differed from the other (all *P* < 0.05), except for comparisons of CA- and HCM+ (*P* = 0.82). In CA and HCM subgroups, GLevNonU exhibited a stepwise elevation (CA-vs. CA+, *P* = 0.006) (HCM- vs. HCM+, *P* < 0.001), HCM- had significantly elevated GLevNonU, compared to both CA- and normal controls (*P* = 0.02, *P* = 0.003) (Figure 5 and Supplementary Table 2).

### Diagnostic performance of radiomics-magnetic resonance combined model

Finally, the combined model was constructed using four conventional metrics and one texture feature (i.e., Maximum LVWT, Asymmetric septal hypertrophy, LV mass, LGE, and GLevNonU). The efficiency of the combined model to classify CA and HCM was evaluated: The AUC, sensitivity, and specificity were 0.89 (CI: 0.85–0.94), 0.80 (CI: 0.70–0.87), and 0.85 (CI: 0.76–0.91) in the validation group, respectively.

### Comparison of diagnostic performance of different models

**FIGURE 6:**
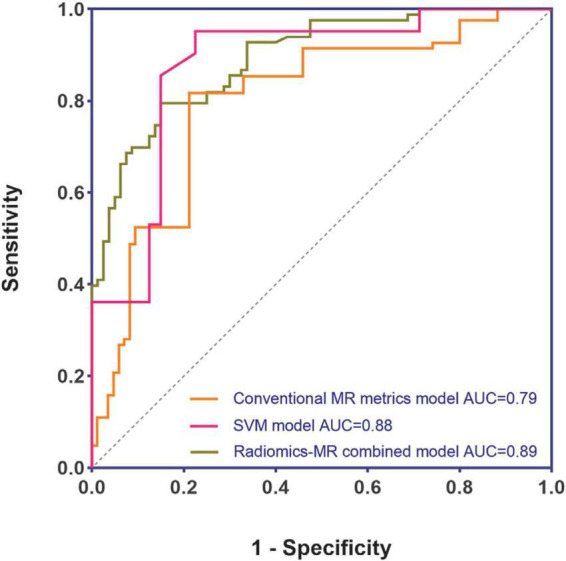
Performance comparison of different models.

Based on the validation group, the highest AUC (0.89), accuracy (0.83), and specificity (0.85) were found in the radiomics-imaging combined model to classify CA and HCM with sensitivity (0.80). Similarly, the SVM model to classify CA and HCM also had the AUC (0.88) in the validation group. No statistically significant difference was observed between the two AUCs according to the DeLong’s test (*P* = 0.62) (Figure 6).

## Discussion

The main findings of this study are as follows: In a clinical cohort of CA and HCM patients, we differentiated between these two diseases on non-contrast cine CMR images using machine learning-based MRI classifiers, which achieved a satisfactory performance. The best model was SVM classifiers, and the most contributing texture feature for classification was GLevNonU. A previous study differentiated the etiologies of left ventricular hypertrophy based on first-order statistics histogram from SAX cine images. Myocardial involvement between HCM and CA was found to differ (20). To the best of our knowledge, our study is the first to establish ML-based classifiers that combine TA to distinguish between CA and HCM on non-contrast cine images.

Cine images routinely provide the initial diagnostic impression in classifying non-ischemic cardiomyopathy based on different patterns of left ventricular hypertrophy (LVH). LGE imaging further improves diagnostic confidence. However, conventional radiological features visually assessed in the clinical routine are not always typical; hence, they can vary based on a radiologist’s subjective opinion (6, 17). Radiomics is a potential tool in modern radiology. It can overcome the limitations of visual assessment and provide a quantitative objective estimation of tissue features. By extracting and analyzing a large number of image features that cannot be detected by the naked eye, it can provide important information on histopathology.

Advanced imaging tools including T1 and extracellular volume (ECV) mapping, are now widely used for differential diagnosis of LVH patients because they overcome the limitations of conventional radiological feature classification. Based on T1 mapping images, Neisius et al. (21) reported a maximum diagnostic accuracy of 86.2%. Martini et al. (22) investigated the diagnosis of CA from CMR using deep learning. Advanced imaging is not always available for all medical conditions and it requires sophisticated acquisition and analysis techniques. This would facilitate clinical work-up if conventional sequences can be used to classify LVH (e.g., cine). To date, only one study has adopted MaZda to extract texture features from non-contrast T1-weight images to perform ML-based classification of HCM. In this study (10), one classifier was trained to differentiate HCM from the control achieving an AUC of 0.95. Recently, the LASSO method was found to be an effective and efficient method for feature selection (7). We used several ML classifiers and feature selection algorithms to identify and explore differences between CA and HCM, and various trained classifiers were used, including: ANN, SVM, k-NN, NB, LR, and RF. Before verification, each classifier was subjected to internal cross-verification to evaluate the classification accuracy and avoid overfitting.

The best model was SVM classifiers. Validation data showed a decrease in accuracy of the selected features, as is common in cardiac “omics” studies (23). Compared with other ML methods, SVM was found to be suitable for identifying subtle patterns in complex data sets (24, 25). It has also been proven to play a crucial role in cancer classification and sub-classification in oncogenomics. Vamvakas et al. (26) used SVM to classify low-grade and high-grade gliomas with TA. With a leave one out cross validation (LOOCV), they achieved an accuracy of 0.955 and an AUC of 0.955.

Radiomics models are slowly gaining traction as superior diagnostic tools to conventional image analysis. Neisius et al. (21) applied exhaustive texture analysis using six radiomics features and a SVM model combined also showing an improved accuracy of 0.86. Unlike this study, in our study, the most contributing radiomics feature was GLevNonU, and we found that radiomics and conventional MR image metrics can be combined to improve the differential diagnosis performance, and the combined model had a significant higher AUC (0.89) than the conventional MR imaging model (0.79). It should be noted that the combined conventional CMR metric model already utilizes and benefits from LGE information. In contrast, radiomics model building uses only radiomics features obtained from non-contrast images. Radiomics can derive quantitative metrics on a number of aspects, including tissue shape and texture, by analyzing pixel-level data (27). The type of information provided by radiomics conceptually suggests that features not captured by current conventional image analysis methods can be extracted from existing CMR images by means of radiomics, and that this information appears to provide additional insight into myocardial microstructural remodeling patterns (28) so that models combining both radiomics and conventional imaging graphics parameters can be used to improve diagnostic performance. Previous studies (29, 30) in other fields have also shown the combined model performs better than single conventional imaging or radiomics model.

For detecting myocardial tissue alterations, the most important texture feature was GLevNonU from GLRLM in our study. As a widely used texture analysis algorithm, GLRLM is determined by computing the number of gray level runs, where gray level runs correspond to a set of linearly adjacent picture points of the same gray-level value (10). GlevNonU measures pixel intensity diversity along a line toward the horizontal, thus, higher values indicate more significant inhomogeneity at the pixel level in the myocardium. Myocardial heterogeneity with concomitant alterations of GLevNonU have been shown on T1-sequences in HCM and T2 mapping in myocarditis (10, 31). Our results showed that GLevNonU on cine-based images was significantly higher in CA patients relative to controls, which supports the hypothesis that microstructures of the myocardium of HCM patients may have a heterogeneous tissue texture. This is possible considering that myocardial tissue heterogeneity associated with GLevNonU alterations has also been identified in other myocardial diffuse diseases (10, 32). we compared GLevNonU in CA and HCM and its ability to differentiate them. Using non-enhanced cine images, we found that GLevNonU in HCM was higher than in CA and controls. Even in the equal ASH subgroup, the GLevNonU value in HCM was markedly higher relative to CA patients with a similar degree of LVWT.

The myocardial structural pattern differs between HCM and CA. Unlike CA, HCM is characterized by myocyte hypertrophy, disarray, and diffuse interstitial and perivascular fibrosis. Case of infiltrative cardiomyopathy like CA present with enlarged extracellular space with slight morphologic changes of myocytes (5). Therefore, we postulated that GLevNonU might be more sensitive to morphological or structural changes of myocytes than extracellular changes. Several tumor-related histological studies had also similar findings with us: GLevNonU was an essential feature, which was attributed due to the significant morphological and structural differences between tumor cells and normal cells (33). Conventional cine imaging cannot directly assess myocardial microstructural abnormalities, including hypertrophic disarray of cardiomyocytes. However, it can be detected by TA. Thornhill et al. (34) found that GLevNonU differentiated LGE+ from LGE-myocardium of HCM patients as well as between HCM patients and healthy controls, suggesting that GLevNonU may identify incipient myocardial markers and microstructural abnormalities in HCM patients. In HCM patients, Cui et al. (35) found good correlations between LGE and collagen and, therefore, fibrosis, but not with myocardial disarray. Accordingly, one might consider whether GLevNonU and/or other texture features can detect different aspects of myocardial damage (e.g., myocyte hypertrophy and disarray), compared with LGE, which is directly linked to ventricular arrhythmias (35).

It should be noted that even in the LGE- subgroup, GLevNonU still showed a significant increase in HCM-subgroup, compared with CA- subgroup (*P* = 0.02) and controls (*P* = 0.001). LGE location and extent are of particular value in differential diagnosis of non-ischemic cardiomyopathy. Negative LGE complicates diagnosis, which makes it more challenging. Consequently, GLevNonU may provide a more sensitive marker of myocardial microstructure abnormality. Therefore, TA is an effective diagnosis took and may complement the LGE technique for potential clinical application, the two techniques also might be used complementary in future studies.

Applying model-based machine learning to decode various statistical outputs from TA is becoming more widely used, yielding clinically relevant and valuable achievements. Radiomics has several advantages. This technology, which can be retrospectively performed on basically any acquired image, does not require additional scanning time, sequences, or gadolinium-based contrast agents. An intensity-based radiomic features approach have great potential for identifying disease accurately and provide insight into disease process at the tissue level. Consequently, radiomics has real potential to become a very high yield, complementary image analysis tool in clinical settings.

This study has several limitations. First, it was a single-center study with a relatively small sample size, without external validation, thus, a larger sample-sized multicenter study is needed for validation in future clinical applications; Second, only cine-MR was analyzed. Compared with novel sequences, such as T1,T2 and ECV mapping, the information provided by conventional sequences about the microstructure and heterogeneity of myocardium is limited. A third potential limitation is that radiomics features also suffer from lack of robustness as they have been shown to change with imaging parameters, ROI contouring and imaging equipment.

## Conclusion

Machine learning methods can reliably differentiate CA from HCM based on non-contrast cine sequence data. TA may be used to assess myocardial microstructure changes that occur during different stages of cardiomyopathies. TA combined with ML might play an important role in clinical assessment of cardiomyopathy.

## Data availability statement

The raw data supporting the conclusions of this article will be made available by the authors, without undue reservation.

## Ethics statement

The studies involving human participants were reviewed and approved by the Ethics Committee of the First Affiliated Hospital of Soochow University (Approval No. 2017254). The patients/participants provided their written informed consent to participate in this study. Written informed consent was obtained from the individual(s) for the publication of any potentially identifiable images or data included in this article.

## Author contributions

CH was the guarantor of the article. SJ, LZ, and JW contributed to the conception, design, collection, and assembly of data. SJ, LZ, and YF performed the experimental studies and prepared the first draft of the manuscript, which was critically revised by SH and CH. JW, XL, XW, and SWH contributed to the data analysis and interpretation. All authors contributed to the article and approved the submitted version.

## Footnotes

1
https://www.Eletel.p.Lodz.Pl/mAzda/
