# Supplementary material for: Differentiating between cardiac amyloidosis and hypertrophic cardiomyopathy on non-contrast cine-magnetic resonance images using machine learning-based radiomics
Source: Front Cardiovasc Med. 2022 Oct 26;9:1001269. doi: 10.3389/fcvm.2022.1001269 (PMC9643770; doi:10.3389/fcvm.2022.1001269)
Supplement: Supplementary file 1 [file Data_Sheet_1.docx]

Supplementary Material

# Supplementary Methods

## Cardiac Amyloidosis Cohort Composition

Biopsy of all patients confirmed AL amyloidosis by Congo red stain, immunofluorescence, or mass spectrometry confirmation of light chains deposition. The following tissues were used for the assays: kidney (n = 36), bone marrow (n =12), fat (n = 10), tongue (n = 7), myocardium (n = 5 ) , liver (n = 4 ) , upper gastro intestinal tract (n = 3), buccal mucosa (n = 3), rectum (n = 3) and skin (n = 2). Laboratory examination of cardiac biomarkers was performed on all patients with Troponin I (cTnI) and N-terminal pro-B-type natriuretic peptide (NT-proBNP). The “typical features” include the presence of LVH (defined as end-diastolic thickness of the interventricular septum >12 mm in the absence of hypertension or other potential causes) (1)or LGE was present in a typical pattern (circumferential pattern involving the entire subendocardium, extending to adjacent myocardium) (2) The term, LVH, was synonymous with ‘left ventricular wall thickness’, independent of LV mass.

## Control Cohort Composition

The control group consisted of 7 healthy adult subjects recruited through advertisement and 77 clinical patients screened for any history of cardiac disease. Referral indications for a clinical CMR were exclusion of cardiomyopathies in patients with a known familial fabry (n=6); exclusion of structural heart disease (coronary artery disease, valvular heart disease or congenital heart disease) as part of differential diagnostic processes (n=55), or in the context of abnormal electrocardiograms (n=9), and abnormal/inconclusive echocardiography findings (n=8).

## Cardiac MR Imaging Protocol

The maximum gradient field was 45 mT / m, and the maximum gradient switching rate was 200 mT • m-1 • ms-1. An 18-element body matrix coil and a 32-cell array of spine arrays were used for data collection.

Both Cine and LGE images consisted of 2-chamber, 3-chamber, 4-chamber, and 10-12 short-axis images covering the entire ventricles from base to apex.

Typical parameters were as follows: repetition time (TR)/echo time (TE), 3.3/1.43 ms; flip angle (FA), 55°-70°; voxel size, 1.6x1.6x6.0 mm3; temporal resolution, 45.6 ms; and bandwidth, 962 Hz/pixel.)

## Reasons for the ROIs placement

Alis et al (3) reported radiomics features of CMR cine images greatly vary during the cardiac cycle. The radiomics features of end-systolic cine images are more robust than end-diastolic cine images in terms of reproducibility. Accuracy and consistency play a key role in diagnosis. Martini et al (4) found that the comparison of diagnosis performance obtained by analyzing images from different levels of cine sequence shows that 4-chamber long-axis(LAX) images can obtain the most informative features, the loss of training and verification subset is the smallest, and the diagnosis performance and accuracy are the highest. However, the previous works investigating the radiomics features of CMR cine images only at the mid-ventricular level(5–9), the 4ch image position is relatively fixed, and it can take into account the basal segment, middle segment and apical segment of myocardium; hence, the radiomics analysis of LAX cine images seems to be a noteworthy topic to explore in future studies. Cine 4ch LAX images are generally acquired before the contrast administration, while in some institutions, Cine sax images are obtained after contrast injection. To summarize, 4ch LAX image (end-systolic) selected for ROI drawing is appropriate.

## Define of asymmetric hypertrophy (ASH)

In a standard 17-segment model, we recorded maximal and minimal dimensions and their location based on end-diastolic short-axis cine images

An asymmetry ratio was obtained by dividing the maximal by the minimal LV wall thicknesses within the same slice. ASH was considered present if one of the segments was >12 mm in thickness(8) and the asymmetry ratio was≥1.322(11,12)

# Supplementary References

1. Gertz MA, Comenzo R, Falk RH, Fermand JP, Hazenberg BP, Hawkins PN, Merlini G, Moreau P, Ronco P, Sanchorawala V, et al. Definition of organ involvement and treatment response in immunoglobulin light chain amyloidosis (AL): a consensus opinion from the 10th International Symposium on Amyloid and Amyloidosis, Tours, France, 18-22 April 2004. Am J Hematol (2005) 79:319–328. doi: 10.1002/ajh.20381

2. Fontana M, Pica S, Reant P, Abdel-Gadir A, Treibel TA, Banypersad SM, Maestrini V, Barcella W, Rosmini S, Bulluck H, et al. Prognostic Value of Late Gadolinium Enhancement Cardiovascular Magnetic Resonance in Cardiac Amyloidosis. Circulation (2015) 132:1570–1579. doi: 10.1161/CIRCULATIONAHA.115.016567

3. Alis D, Yergin M, Asmakutlu O, Topel C, Karaarslan E. The influence of cardiac motion on radiomics features: radiomics features of non-enhanced CMR cine images greatly vary through the cardiac cycle. Eur Radiol (2021) 31:2706–2715. doi: 10.1007/s00330-020-07370-y

4. Martini N, Aimo A, Barison A, Della Latta D, Vergaro G, Aquaro GD, Ripoli A, Emdin M, Chiappino D. Deep learning to diagnose cardiac amyloidosis from cardiovascular magnetic resonance. J Cardiovasc Magn Reson (2020) 22: doi: 10.1186/s12968-020-00690-4

5. Larroza A, Materka A, López-Lereu MP, Monmeneu JV, Bodí V, Moratal D. Differentiation between acute and chronic myocardial infarction by means of texture analysis of late gadolinium enhancement and cine cardiac magnetic resonance imaging. Eur J Radiol (2017) 92:78–83. doi: 10.1016/j.ejrad.2017.04.024

6. Schofield R, Ganeshan B, Fontana M, Nasis A, Castelletti S, Rosmini S, Treibel TA, Manisty C, Endozo R, Groves A, et al. Texture analysis of cardiovascular magnetic resonance cine images differentiates aetiologies of left ventricular hypertrophy. Clin Radiol (2019) 74:140–149. doi: 10.1016/j.crad.2018.09.016

7. Baeßler B, Mannil M, Maintz D, Alkadhi H, Manka R. Texture analysis and machine learning of non-contrast T1-weighted MR images in patients with hypertrophic cardiomyopathy—Preliminary results. Eur J Radiol (2018) 102:61–67. doi: 10.1016/j.ejrad.2018.03.013

8. Baessler B, Mannil M, Oebel S, Maintz D, Alkadhi H, Manka R. Subacute and Chronic Left Ventricular Myocardial Scar: Accuracy of Texture Analysis on Nonenhanced Cine MR Images. Radiology (2018) 286:103–112. doi: 10.1148/radiol.2017170213

9. Baessler B, Luecke C, Lurz J, Klingel K, von Roeder M, de Waha S, Besler C, Maintz D, Gutberlet M, Thiele H, et al. Cardiac MRI Texture Analysis of T1 and T2 Maps in Patients with Infarctlike Acute Myocarditis. Radiology (2018) 289:357–365. doi: 10.1148/radiol.2018180411

10. Lang RM, Bierig M, Devereux RB, Flachskampf FA, Foster E, Pellikka PA, Picard MH, Roman MJ, Seward J, Shanewise JS, et al. Recommendations for chamber quantification: a report from the American Society of Echocardiography’s Guidelines and Standards Committee and the Chamber Quantification Writing Group, developed in conjunction with the European Association of Echocardiography, a branch of the European Society of Cardiology. J Am Soc Echocardiogr Off Publ Am Soc Echocardiogr (2005) 18:1440–1463. doi: 10.1016/j.echo.2005.10.005

11. Maron BJ. Asymmetry in hypertrophic cardiomyopathy: the septal to free wall thickness ratio revisited. Am J Cardiol (1985) 55:835–838. doi: 10.1016/0002-9149(85)90166-3

12. Afonso LC, Bernal J, Bax JJ, Abraham TP. Echocardiography in hypertrophic cardiomyopathy: the role of conventional and emerging technologies. JACC Cardiovasc Imaging (2008) 1:787–800. doi: 10.1016/j.jcmg.2008.09.002

# Supplementary Figure

## Supplementary Figure S1


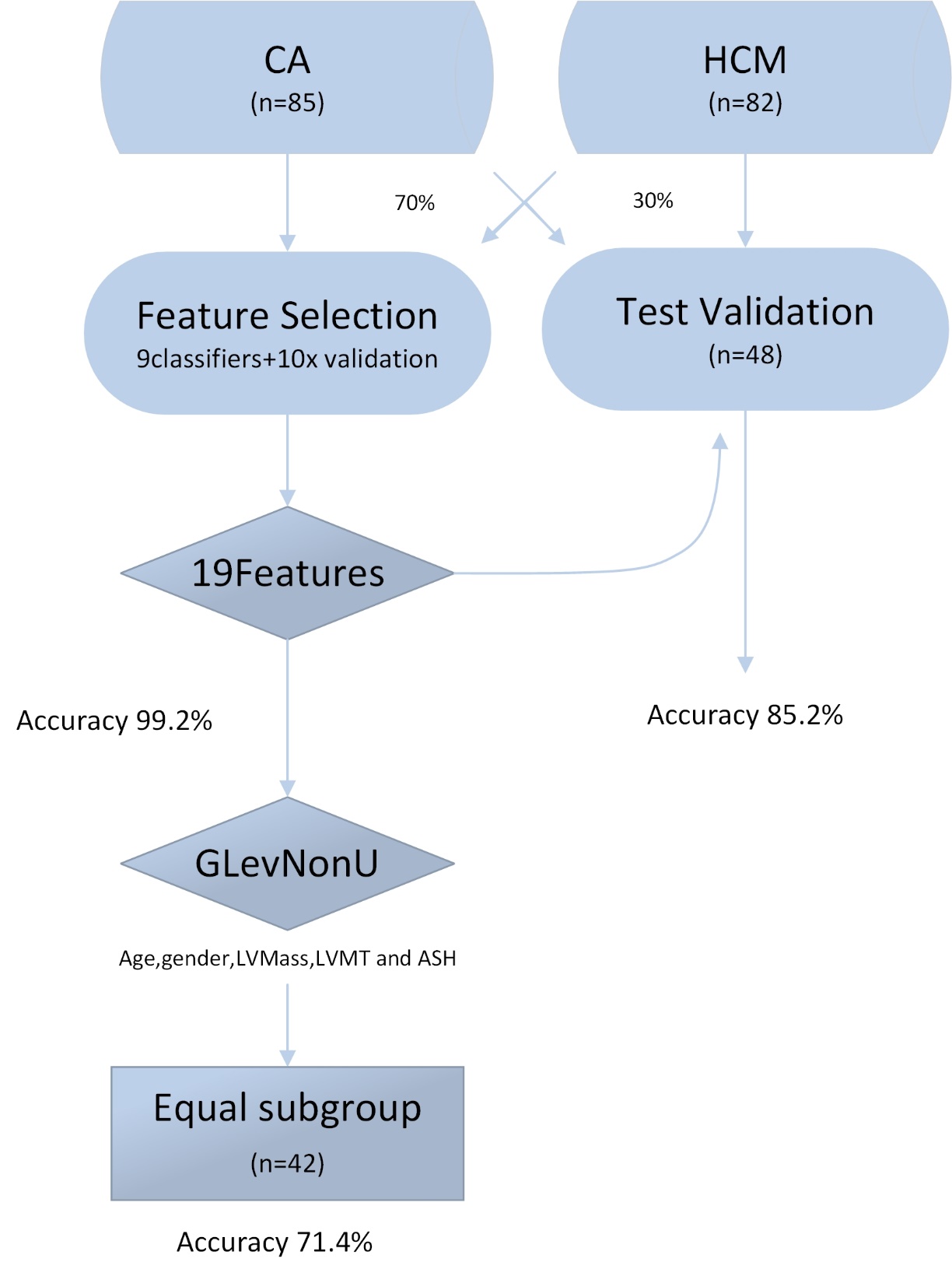


**Figure S1.** Flow diagram of the CINE image texture analysis plan. Illustrated are the division into feature selection and test validation datasets, as well as test accuracies for the selected tissue features. LVWT, left ventricular wall thickness; ASH: asymmetric septal hypertrophy;LGE: late gadolinium enhancement.

## Supplementary Figure S2


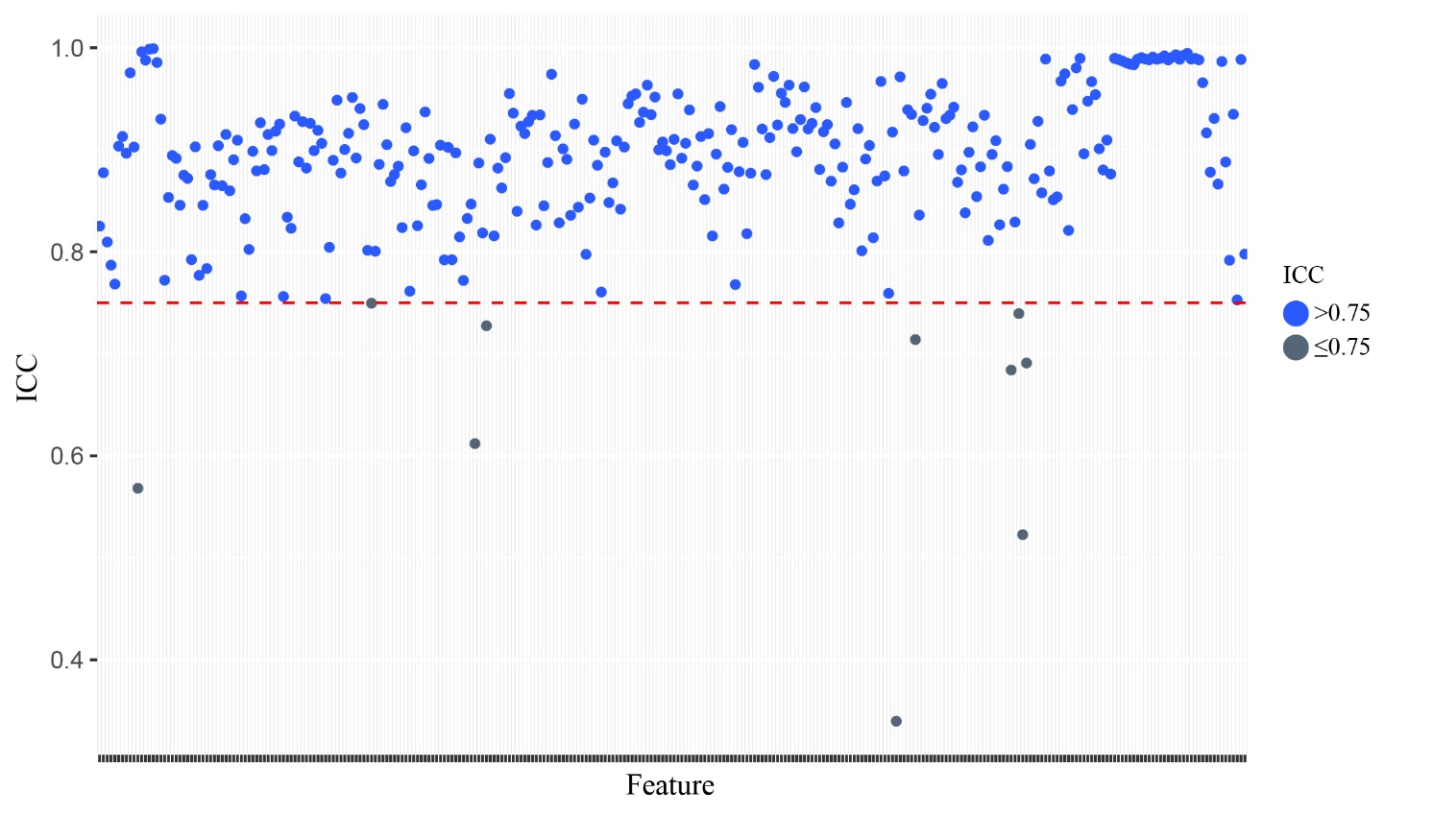


**Figure S2.** The Intraclass Correlation Coefficient (ICC) is given for scatter plot and horizontal lines are inserted to denote ICC cut off at 0.75.

## Supplementary Figure S3


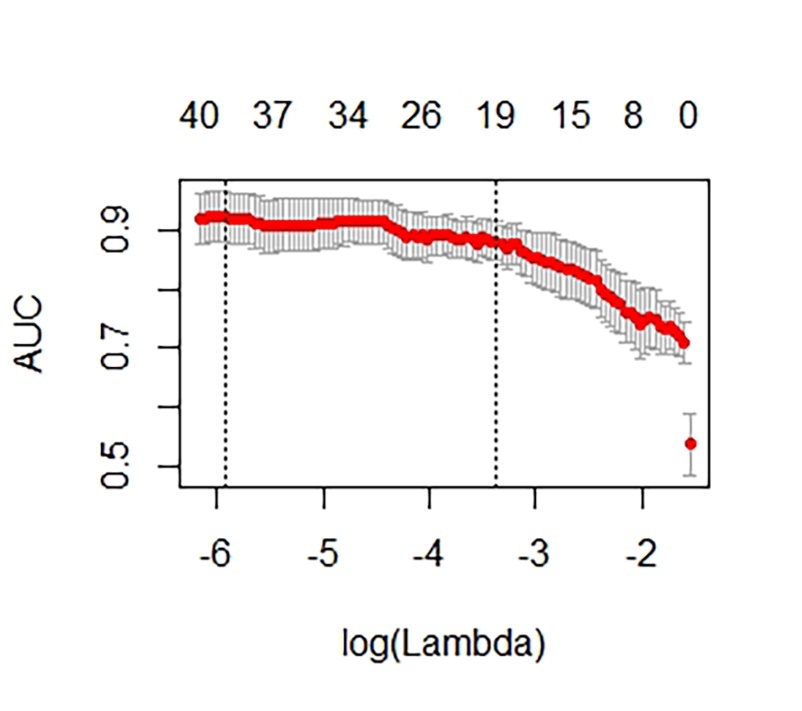
(a)


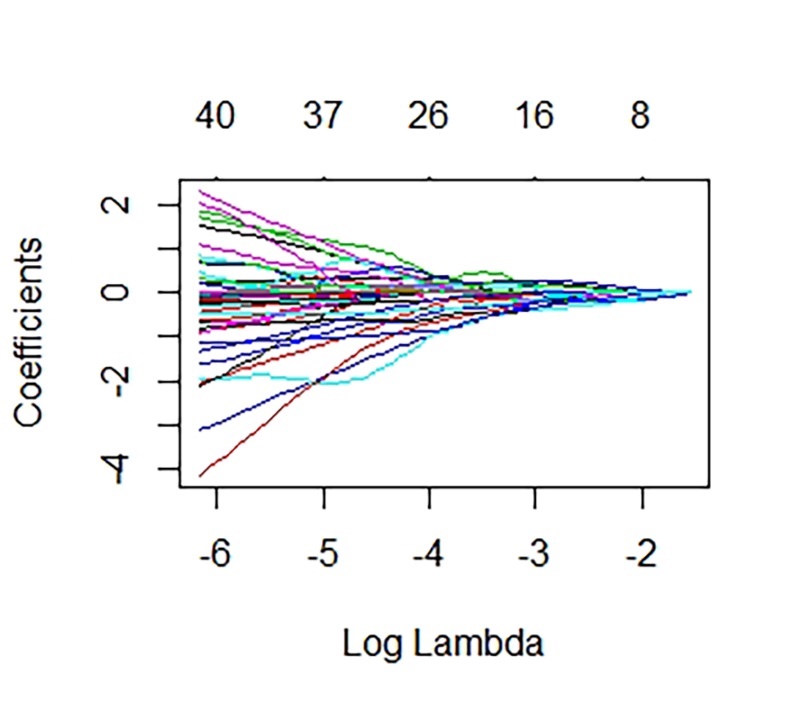
(b)

**Figure S3.** Lasso regression analysis results: (a) The area under the curve (AUC) for the lasso regression; (b) Lasso regression analysis.

## Supplementary Figure S4

**
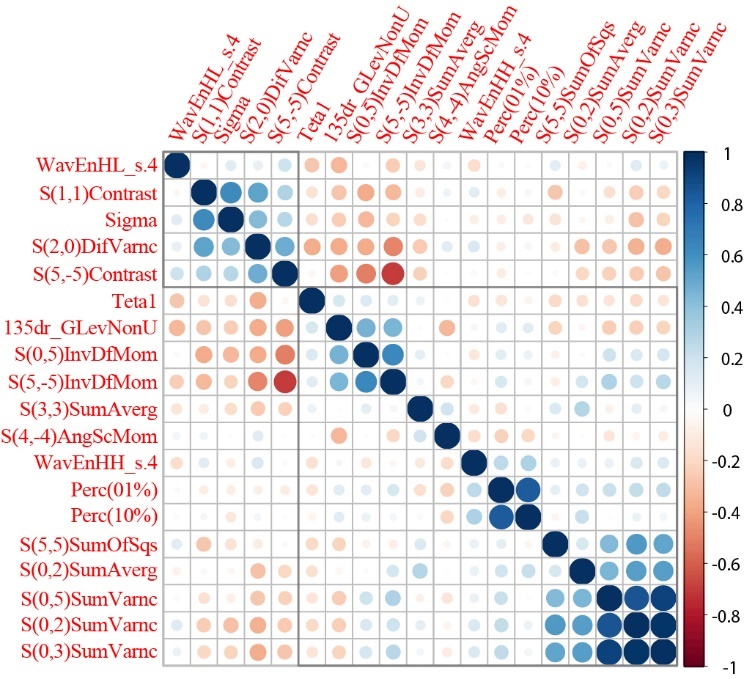
**

**Figure S4.** Correlogram illustrating auto- and cross-correlations of the 19 most important texture features. Texture features were reordered after hierarchical clustering for visualizing different feature clusters. Blue circles indicate positive correlation, red circles negative correlation. The larger the circle and the darker the colour, the higher is the correlation between two variables.

## Supplementary Figure 5


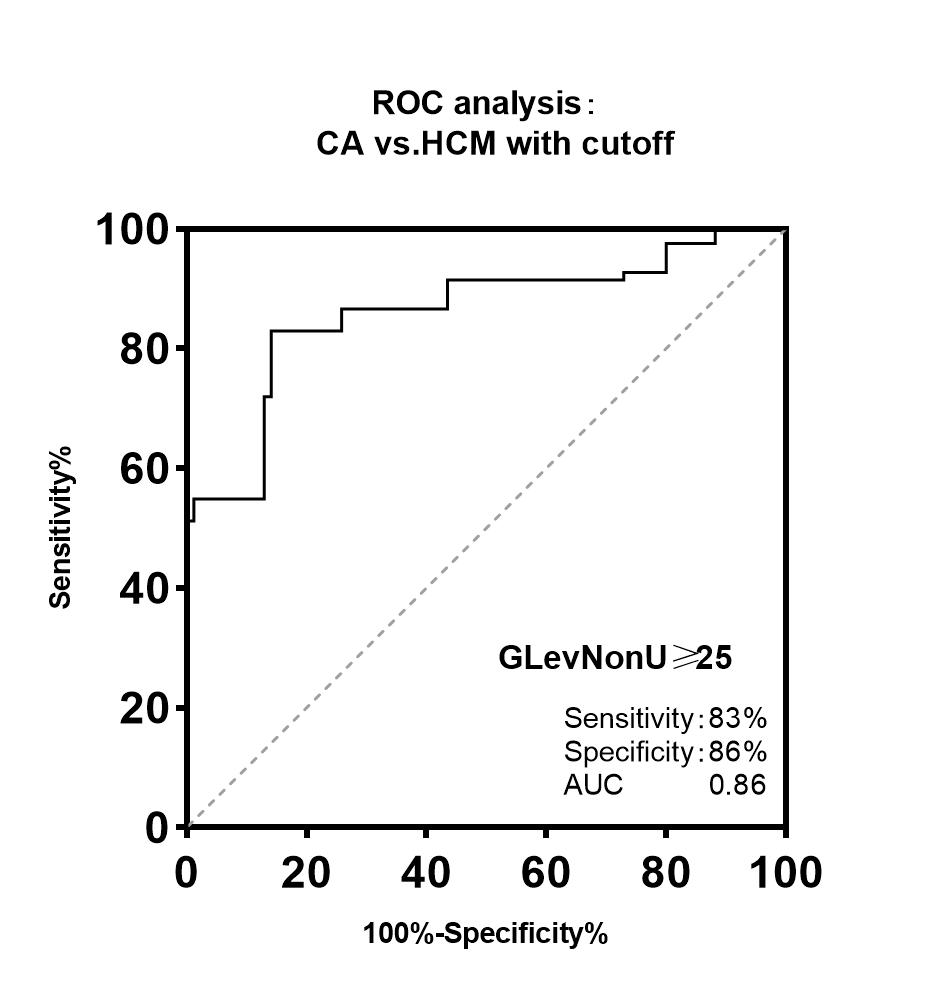
a
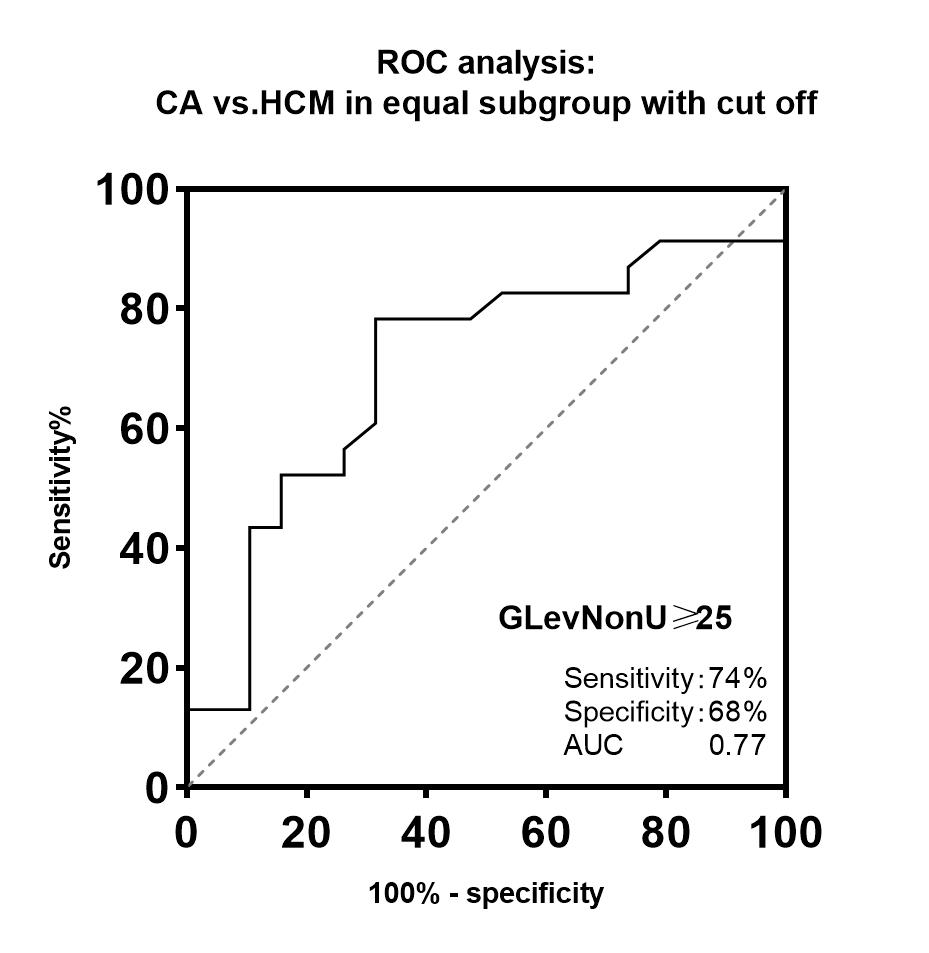
b

**Figure S5.** ROC analysis indicating the accuracy of the texture feature Gray level non-uniformity (GLevNonU) for diagnosing the CA in all patients with cut-off (a) and the equal subgroup with cut-off (b)

# Supplementary Tables

## Table S1. Clinical characteristics of the equal subgroup.

| Parameter | CA  patients(n=19) | HCM  patients(n=23) | *P*-value |
| --- | --- | --- | --- |
| Age (year) | 54±9 | 46±14 | 0.47 |
| Male,n(%)  LVWT ≥15 mm, n (%)  Maximal LVWT,mm | 40(47)  9(49)  14.4±2.0 | 44(54)  12(52)  14.6±1.2 | 0.96  0.85  0.89 |
| LV mass(g/m^2^) | 76.9±18.2 | 75.7.±24.1 | 0.71 |

*CA, cardiac amyloidosis; HCM, hypertrophic cardiomyopathy; LV mass LVWT, left ventricular wall thickness. Continuous variables are presented as mean ± standard deviation or as median, interquartile range, and 95% confidence interval (CI).* *Categorical variables are presented as n (%).*

## Table S2. Values of GLevNonU in the different subgroups

|  |  | CA-(n=17) | CA+(n=68) | HCM-(n=31) | HCM+(n=51) | Control(n=84) |
| --- | --- | --- | --- | --- | --- | --- |
|  | GLevNonU | 18.71(17.62, 22.29) | 26.12(22.87, 32.45) | 27.12(23.41, 30.60) | 39.55(35.38, 47.24) | 12.24(9.031, 15.50) |
|  | 95% CI | 17.66-22.10 | 24.25-29.04 | 24.49-29.49 | 36.70-42.51 | 12.11-13.70 |

*Abbreviations as Table S1. Data are presented as median, interquartile range, and 95% confidence interval (CI).*
